# Polo-like kinase isoform expression is a prognostic factor in ovarian carcinoma

**DOI:** 10.1038/sj.bjc.6601610

**Published:** 2004-02-17

**Authors:** W Weichert, C Denkert, M Schmidt, V Gekeler, G Wolf, M Köbel, M Dietel, S Hauptmann

**Affiliations:** 1 Institute of Pathology, Charité Hospital, Humboldt University, Schumannstrasse 20/21, Berlin 10117, Germany; 2 Altana Pharma AG, Konstanz, Germany; 3 Institute of Pathology, Martin-Luther-University Halle-Wittenberg, Halle, Germany

**Keywords:** Polo-like kinase, ovarian carcinoma, survival, mitosis

## Abstract

The Polo-like kinase (PLK) family comprises three serine/threonine kinases, functionally involved in signal transduction pathways essential for the accomplishment of mitosis in both normal and malignant cells. Moreover, certain PLKs have been functionally linked to cytoskeletal reorganisation. In this study, the expression of PLK1 and PLK3 was determined immunohistochemically in tissue specimen of normal ovaries (*n*=9), cystadenomas (*n*=17), borderline tumours (*n*=13) and ovarian carcinomas (*n*=77). PLK 1 and PLK3 expression was low in normal ovarian surface epithelium and borderline tumours, with moderately higher expression levels in cystadenomas. In ovarian carcinomas, 26% of cases were PLK1 positive and 50.6% of cases were PLK3 positive. A positive correlation of both PLK1 and PLK3 expression with indicators of mitotic frequency could be established. The overexpression of either isoenzyme had an impact on patient prognosis with shortened survival time for patients with tumours positive for PLK1 (*P*=0.02) and PLK3 (*P*=0.02), but only PLK1 expression remained a prognostic factor in multivariate survival analysis (*P*=0.03). The results of this study, if interpreted in the context of recently published functional data, suggest that inhibition of PLKs might represent an interesting new targeted approach for chemotherapy of epithelial ovarian cancer. Furthermore, this study suggests that PLK1 is a novel independent prognostic marker in ovarian carcinomas.

In most industrialised countries, ovarian cancer is the fifth leading cause of cancer death in women with an overall of 14 300 estimated ovarian cancer-related deaths in the US in 2003 (Jemal *et al*, 2003). More than 90% of ovarian malignancies arise from the ovarian surface epithelium forming the group of epithelial ovarian carcinoma.

Surgery and chemotherapy play a major role in the treatment of ovarian carcinoma, but mortality has changed little over the past 20 years (Edmondson and Monaghan, 2001), indicating that the biology of this tumour entity is still poorly understood. Owing to the fact that ovarian cancer is usually detected at advanced disease stages, chemotherapy is particularly important in these tumours. Most drugs used in ovarian carcinoma treatment are effective in mitotically active cells. Therefore, knowledge on regulation of mitosis is an important step in establishing new treatment strategies for these common tumours.

There is abundant evidence that Polo-like kinase (PLK) isoforms play an important role in a number of intracellular signal transduction pathways related to mitosis (see, for review, Nigg, 1998; Donaldson *et al*, 2001). The expression of members of this kinase family in malignancies is of special interest, because inhibitory strategies for PLKs might hinder tumour cells to accomplish mitosis and subsequently induce tumour cell death and reduce overall tumour growth.

The name of the PLK family stems from *Drosophila* gene *Polo*, where this special type of serine/threonine kinases have first been identified (Sunkel and Glover, 1988). In recent years, varying numbers of Polo homologues have been discovered in a broad variety of organisms, including yeast, certain bacteria, mice and men. The family of human PLK isoenzymes, comprises to date three family members, which are now named PLK1, PLK2 and PLK3 (formerly designated as PLK, SNK and FNK), respectively (Nigg, 1998). Sak, another kinase related to the PLK family comprises only one Polo box, yet has been recently referred to as PLK4 (Manning *et al*, 2002). All isoenzymes share a closely related catalytic domain near the amino-terminal end of the protein and a highly characteristic sequence motif, the so-called Polo boxes near the carboxy-terminal end of the proteins. For PLK1, there is convincing evidence that protein expression and kinase activity peak through G2/M transition and that regular protein function is necessary for the entry into and progression through mitosis by a variety of different functional mechanisms (Donaldson *et al*, 2001).

Studies on the expression of PLK1 have been performed on carcinomas of lung (Wolf *et al*, 1997), head and neck (Knecht *et al*, 1999), oesophagus and stomach (Tokumitsu *et al*, 1999), skin (Kneisel *et al*, 2002), breast (Wolf *et al*, 2000), brain (Dietzmann *et al*, 2001), endometrium and ovary (Takai *et al*, 2001a, 2001b). All studies consistently reported an overexpression of PLK1 in the respective tumour tissue compared to the corresponding nontransformed tissue of origin. In addition, some studies suggested a prognostic role for PLK1 expression.

In contrast to the extensively studied PLK1 isoform, information with respect to the expression, regulation and function of PLK3 is sparse and results are somewhat contradictory. To our knowledge, there are currently no studies available on the expression of PLK isoform proteins other than PLK1 in either normal human tissue or malignant human tumours.

Therefore, the aim of this study was to investigate the expression of PLK1 and PLK3 in epithelial ovarian tumours and to perform an in-depth exploration of the association between PLK isoform expression, clinicopathological data and patient survival.

## MATERIAL AND METHODS

### Patients and tissue samples

Tissue samples were collected from 116 patients (age range: 28–85 years, median 59.5 years) who underwent surgery for diagnostic or therapeutic purpose at the Charité Hospital, Humboldt University, Berlin between 1990 and 2001. The total of 116 samples was subdivided in either normal ovaries (*n*=9), removed for other causes than malignant tumour, benign cystadenomas (*n*=17), borderline tumours (*n*=13) or primary ovarian carcinomas (*n*=77).

None of the patients with malignant tumour received neoadjuvant chemotherapy. Data on postsurgical chemotherapy were available for 50 out of 77 patients with primary carcinomas, of whom 41 (82%) received a platinum-based first-line chemotherapy. The remainder received either no chemotherapy at all (six cases, 12%) or other types of chemotherapy (three cases, 6%).

According to histology, the group of cystadenomas was further subdivided into either serous type (*n*=11) or mucinous type (*n*=6). The subgroup of borderline tumours was classified as either serous (*n*=10), mucinous (*n*=2) or transitional cell (*n*=1) type. The group of malignant tissue comprises all major tumour types of ovarian carcinoma, in detail 41 serous carcinomas, 13 undifferentiated carcinomas, 10 endometrioid carcinomas, six mucinous carcinomas, four clear-cell carcinomas and three transitional cell carcinomas were investigated. Diagnosis, tumour type and histopathological tumour grading (according to the Silverberg grading system of epithelial ovarian tumours) of each sample was confirmed on standard H&E sections by one of the authors (SH). Correlation analysis of PLK1/PLK3 staining with clinicopathological data and patient prognosis was performed, including only data of patients with primary ovarian carcinomas.

### Immunohistochemistry

For immunohistochemical detection of PLK isoenzymes, monoclonal mouse antibodies against PLK1 (BD Transduction laboratories, San Diego, CA, USA.) and PLK3 (BD Transduction laboratories) were used on 5 *μ*m thick paraffin sections. For both antibodies specificity has been tested in preceding studies (Bahassi el *et al*, 2002; Wang *et al*, 2002). For antigen retrieval, slides were placed in 0.01 M sodium citrate buffer, pH 6.0 and boiled for 10 min in a pressure cooker. After several rinses in phosphate-buffered saline (PBS) and pretreatment with blocking medium (DAKO, Glostrup, Denmark) for 10 min, slides were incubated with primary antibody diluted 1 : 500 (PLK1/PLK3) in PBS for 1 h at room temperature and then at 4°C overnight. After washing slides in PBS, bound antibody was detected by a streptavidin–biotin system according to a standard protocol with standard antibody dilutions as provided by the manufacturer (BioGenex, San Ramon, CA, USA). For colour development, a fast red system (Sigma, Deisenhofen, Germany) was applied. After colour development was stopped, slides were cover slipped using PBS/glycerol.

For evaluation of the proliferation index, the expression of KI-67 was assessed on tissue samples by using a monoclonal mouse antibody (DAKO) directed against human KI-67 protein on 5 *μ*m thick paraffin sections. To improve antigen retrieval, the slides were cooked for 10 min as described above. Slides were incubated with primary antibody diluted 1 : 1000 in PBS with 5% BSA for 1 h at room temperature. Bound antibody was detected by using an LSAB-Kit (DAKO) with standard protocol as supplied by the manufacturer. Slides were viewed in a Leica DMRB microscope.

### Evaluation of immunohistochemical tissue staining

Staining of all slides was evaluated independently by two pathologists (WW and CD), who were blinded towards patient characteristics and outcome. For semiquantitative evaluation, an immunoreactivity-scoring (IRS) system was applied. Intensity of staining was designated as either not existent (0), weak (1), moderate (2) or strong (3). The number of cells stained was scored as either no cells stained (0), less than 10% of cells stained (1), 10–50% of cells stained (2), 50–80% of cells stained (3) or more than 80% of cells stained (4). The IRS was calculated by multiplication of these two variables. For statistical analysis, cases were grouped as either PLK negative (IRS 0–6) or PLK positive (IRS 7–12).

### Statistical analysis

Statistical correlation between several clinicopathological factors and expression of PLK was assessed using either *χ*^2^ test for trends, Fisher's exact test or Spearman's test for rank-order correlation. The probability of differences in overall survival as a function of time was determined using a Kaplan–Meier analysis, with probing of significance by applying a log-rank test. Multivariate probing for significance was performed with the Cox's proportional-hazard model. Generally, *P*-values smaller than 0.05 were considered significant. For all statistical procedures SPSS v10.0 software was used.

## RESULTS

### Expression of PLK isoforms in ovarian tissue

The expression of PLK1 was determined in nontransformed ovarian tissue and could not be observed in any normal ovarian surface epithelium investigated, nor did ovarian stroma show PLK1 positivity. Inclusion cysts of ovarian surface epithelium were consistently PLK1 negative. Follicular cysts, present in some but not all ovaries, showed strong staining for PLK1 in theca externa cell layer and served as internal positive control. Corpora lutea showed focal PLK1 positivity.

In cystadenomas, considerable expression of PLK1 was observed in three out of 17 cases (17.6%). Expression was pronounced in papillary areas. Despite weak focal positivity in the majority of borderline tumours, they were all scored negative. In contrast, 20 out of 77 primary ovarian carcinomas (26%) displayed strong cytoplasmic PLK1 expression (Table 1

**Table 1:** Distribution of PLK1 and PLK3 expression in normal ovaries, cystadenomas, borderline tumours and primary ovarian carcinomas

	**Primary carcinomas (*n*=77)**	**Borderline tumours (*n*=13)**	**Benign cystadenomas (*n*=17)**	**Normal ovaries (*n*=9)**
*PLK1*				
Negative (IRS 0–6)	57 (74%)	13 (100%)	14 (82.4%)	9 (100%)
Positive (IRS 7–12)	20 (26%)	0 (0%)	3 (17.6%)	0 (0%)
				
*PLK3*				
Negative (IRS 0–6)	38 (49.4%)	11 (84.6%)	12 (70.6%)	8 (88.9%)
Positive (IRS 7–12)	39 (50.6%)	2 (13.4%)	5 (29.4%)	1 (11.1%)

PLK=Polo-like kinase; IRS=immunoreactivity-scoring system.

, Figure 1

**Figure 1:**
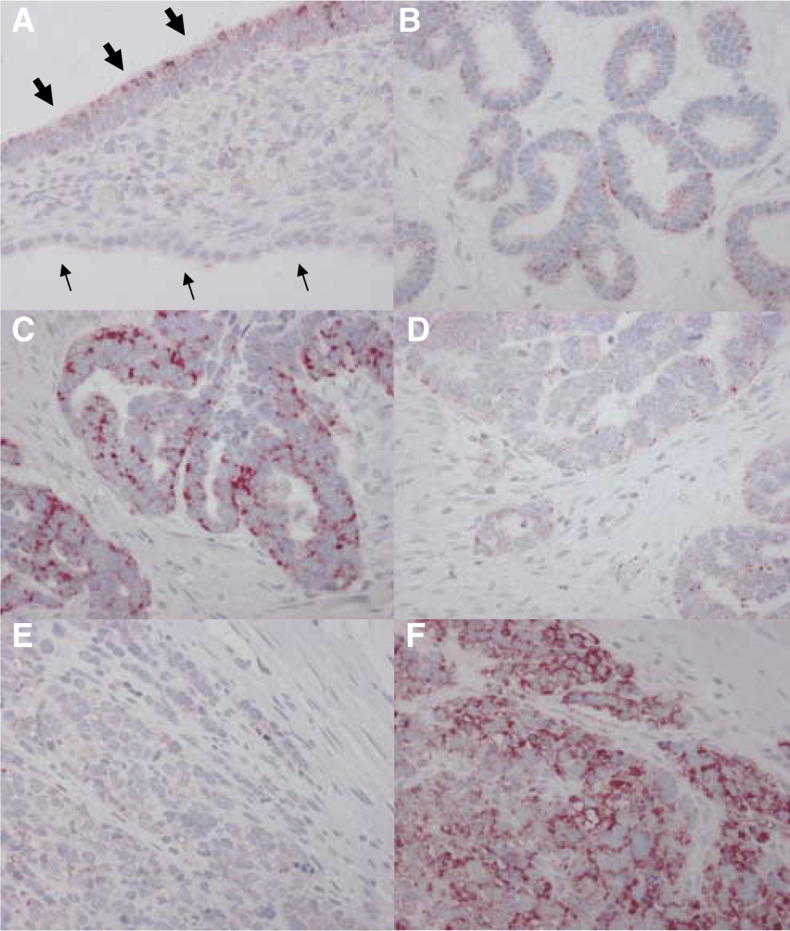
Expression of PLK1 and PLK3 in ovarian tissue specimen. (**A**) Normal ovarian surface epithelium without significant PLK1 positivity (small arrows) and moderate PLK1 positivity in an adjacent serous cystadenoma (bold arrows). (**B**) Serous borderline tumour of the ovary showing only weak scattered expression of PLK3. This tumour was scored as PLK3 negative. (**C**, **D**) Serous ovarian carcinoma with strong expression of PLK1 in more than 80% of tumour cells (**C**), while the same tumour showed only low expression for PLK3 (**D**). (**E**, **F**) Undifferentiated ovarian carcinoma staining strongly positive for PLK3 (**F**) but not for PLK1 (**E**).

). Nevertheless, minor cytoplasmatic staining in a subpopulation of tumour cells could be observed in the epithelium of all tumours. In those cases in which notable PLK1 expression was present and in which infiltrative tumour margins could be seen, PLK1 expression was accentuated on the leading edge of tumour invasion.

PLK3 expression was not detectable in the epithelium of eight (88.9%) out of nine normal ovaries investigated. In one case, immunoreactivity for PLK3 was detected in infoldings of the ovarian surface epithelium but was negative for the surface epithelium. A minor fraction of inclusion cysts (approximately 10%), present in normal ovaries, showed notable focal expression of PLK3. Theca externa of follicular cysts as well as corpora lutea expressed PLK3 strongly and homogeneously.

Cystadenomas of the ovary showed PLK3 positivity in five out of 17 cases (29.4%). Expression was pronounced in papillary tumour areas. In borderline tumours, there was considerable PLK3 positivity in two out of 13 cases (13.4%), although all cases showed scattered focal PLK3-positive cells. In the group of primary ovarian carcinomas, 39 out of 77 cases (50.6%) were PLK3 positive (Table 1, Figure 1), usually with pronounced PLK3 expression along the infiltrative tumour margins. As mentioned for PLK1, nearly all PLK3-negative tumours expressed inhomogeneously small, albeit detectable amounts of cytoplasmatic PLK3 protein. In the stroma of normal ovaries as well as in tumour stroma single inflammatory cells showing strong PLK3 expression were occasionally observed.

### Correlation of PLK1/PLK3 expression with clinicopathological factors

For PLK1 and PLK3 expression, statistical correlation analysis with several clinicopathological factors was performed including all cases of primary ovarian carcinoma (*n*=77).

**Figure 2:**
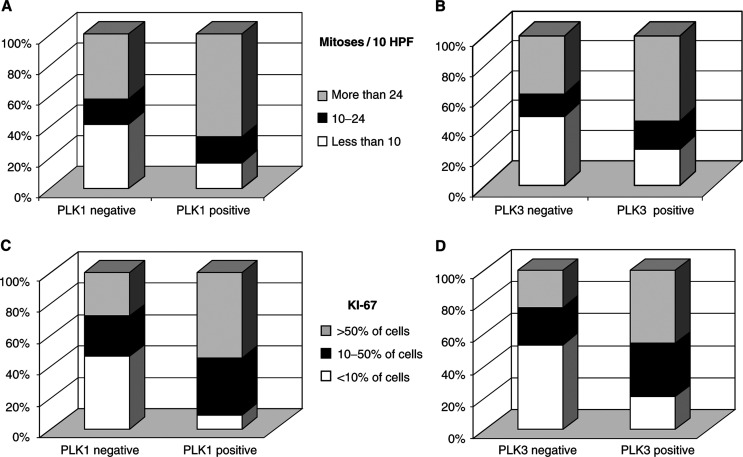
Polo-like kinase isoform expression and correlation to indicators of mitotic frequency. (**A**, **B**) Diagrams showing the distribution of PLK1 expression (**A**) and PLK3 expression (**B**) in dependence of mitotic figure count in the respective tumours. (**C**, **D**) Distribution of KI-67 index in a subgroup of PLK1-positive/negative carcinomas (**C**) and PLK3-positive/negative carcinomas (**D**) (*n*=54).

Ungrouped PLK1 as well as PLK3 expression scores were found to correlate with moderate strength to mitotic figure count (Figure 2

). Correlation coefficient as determined by Spearman's test for rank-order correlation reached 0.229 for PLK1 (*P*=0.044) and 0.254 for PLK3 (*P*=0.026), respectively. In a subgroup of ovarian carcinomas (*n*=54) in which data on KI-67 expression were available, a significant positive correlation of PLK1/PLK3-positive tumours towards higher KI-67 indices in comparison to PLK1/PLK3-negative tumours was observed (Figure 2). *P*-values for these correlations, calculated by the *χ*^2^ test for trends, reached 0.027 for PLK1 and 0.019 for PLK3, respectively.

Usually, the percentage of PLK1/PLK3-positive cells was higher than the KI-67-positive proliferation fraction, so that there was no strict coexpression.

No correlation was observed between PLK1 (*P*=0.165) and PLK3 (*P*=0.289) expression and FIGO stage (Tables 2

**Table 2:** Correlation of PLK1 expression with several clinicopathological factors

	**All cases**	**PLK1 negative (IRS 0-6)**	**PLK1 positive (IRS 7-12)**	***P*-value**
*Age*				
<60 years	42 (54.5%)	30 (71.4%)	12 (28.6%)	0.611+
>60 years	35 (45.5%)	27 (77.1%)	8 (22.9%)	
				
*FIGO stage*				
I	17 (22.1%)	15 (88.2%)	2 (11.8%)	
II	9 (11.7%)	6 (66.7%)	3 (33.3%)	0.165*
III	46 (59.7%)	33 (71.7%)	13 (28.3%)	
IV	5 (6.5%)	3 (60%)	2 (40%)	
				
*Grade*				
G1	15 (19.5%)	13 (86.7%)	2 (13.3%)	
G2	33 (42.9%)	25 (75.8%)	8 (24.2%)	0.125*
G3	29 (37.6%)	19 (65.5%)	10 (34.5%)	

+ Fisher's exact test;

* *χ*^2^ test for trends. PLK=Polo-like kinase; IRS=immunoreactivity-scoring system.

and 3

**Table 3:** Correlation of PLK3 expression with several clinicopathological factors

	**All cases**	**PLK3 negative (IRS 0-6)**	**PLK3 positive (IRS 7-12)**	***P*-value**
*Age*				
<60 years	42 (54.5%)	22 (52.4%)	20 (47.6%)	0.649+
>60 years	35 (45.5%)	16 (45.7%)	19 (54.3%)	
				
*FIGO stage*				
I	17 (22.1%)	9 (52.9%)	8(47.1%)	
II	9 (11.7%)	5 (55.6%)	4 (44.4%)	0.289*
III	46 (59.7%)	24 (52.2%)	22 (47.8%)	
IV	5 (6.5%)	0 (0%)	5 (100%)	
				
*Grade*				
G1	15 (19.5%)	12 (80%)	3 (20%)	
G2	33 (42.9%)	14 (42.4%)	19 (57.6%)	0.025*
G3	29 (37.6%)	12 (41.4%)	17 (58.6%)	

+ Fisher's exact test.

* *χ*^2^ test for trends. PLK=Polo-like kinase; IRS=immunoreactivity-scoring system.

). There was a significant positive correlation for PLK3 (*P*=0.025) but not for PLK1 (*P*=0.125) expression with histopathological tumour grade, high-grade tumours being significantly more likely to express PLK3 than low-grade tumours (Tables 2 and 3).

**Figure 3:**
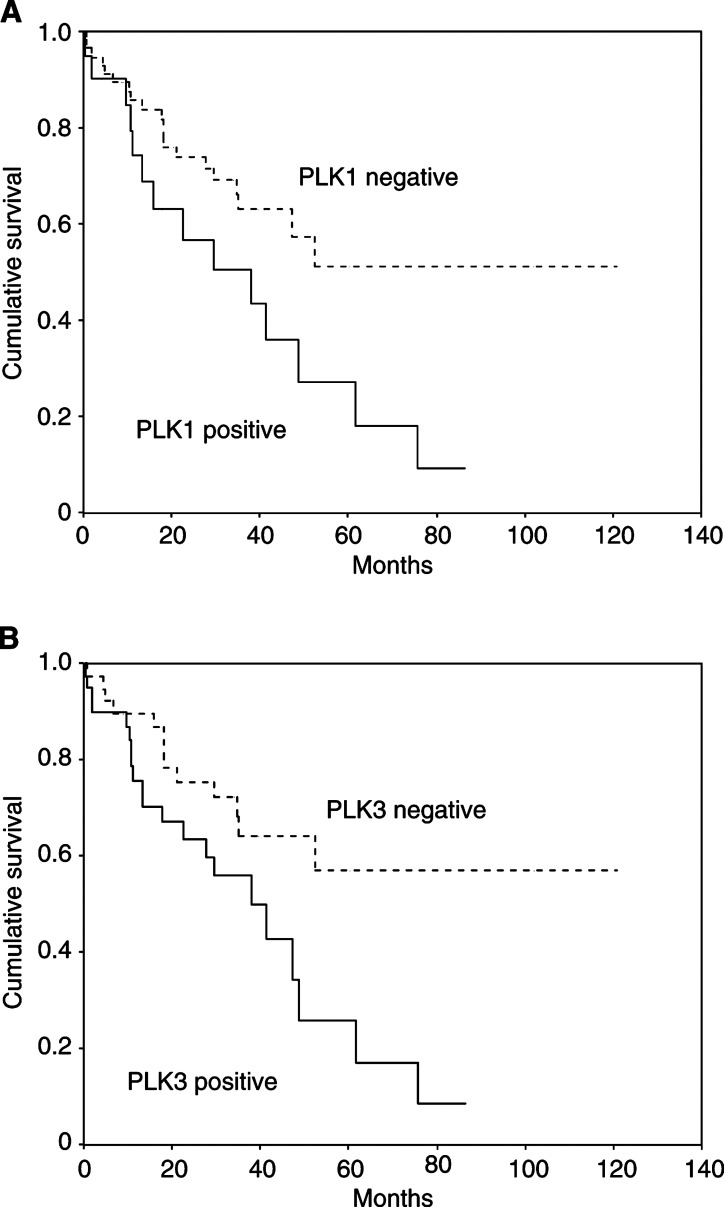
Correlation between patient survival and expression of PLK1 or PLK3. Kaplan–Meier curves for patients grouped as either PLK1 positive or PLK1 negative (**A**) and PLK3 positive or PLK3 negative (**B**). Respective *P*-values in log-rank test for significant differences in survival time were *P*=0.022 for PLK1 and *P*=0.021 for PLK3.

PLK1 expression as well as PLK3 expression had a significant impact on patient prognosis in univariate survival analysis (Figure 3

). Median survival time for patients whose carcinomas were PLK1 or PLK3 negative was not reached, while median survival time for patients with PLK1- and PLK3-positive carcinomas in either groups was 37.9 month (log-rank test for PLK1: *P*=0.022; log-rank test for PLK3: *P*=0.021). The median survival rate after 24 month of follow-up was 52.9% for PLK1-positive cases *vs* 70.2% for PLK1-negative cases and 59.4% for PLK3-positive cases *vs* 72.7% of PLK3-negative cases, respectively. Univariate survival analysis for other clinicopathological tumour features, known to be prognostic factors in ovarian carcinomas revealed a significant prognostic relevance for patient's age at diagnosis (*P*<0.01), FIGO stage (*P*<0.01) and Silverberg grading (*P*<0.01), respectively (Table 4

**Table 4:** Correlation of several clinicopathological factors and of PLK1/PLK3 expression with patient survival (log-rank test)

	**Median survival (month)**	**Standard error**	**Log-rank test (*P*-value)**
*Age at diagnosis*			
<60 years	75.90	20.58	0.0009
>60 years	22.53	6.19	
			
*FIGO stage*			
I	Not reached	—	
II	52.47	29.67	0.0001
III	41.23	7.52	
IV	1.7	0.99	
			
*Grade*			
G1	Not reached	—	0.0015
G2	41.23	7.96	
G3	35.03	5.24	
			
*PLK1*			
Negative	Not reached	—	0.0222
Positive	37.90	13.91	
			
*PLK3*			
Negative	Not reached	—	0.0207
Positive	37.90	8.45	

PLK=Polo-like kinase; IRS=immunoreactivity-scoring system.

). In multivariate survival analysis, under the inclusion of all factors that had a significant impact on patient prognosis in univariate survival analysis, PLK3 expression failed to show independent prognostic significance (*P*=0.49). In contrast, PLK1 expression remained a significant prognostic factor in multivariate survival analysis (Table 5

**Table 5:** Multivariate survival analysis by the Cox's proportional-hazard model for PLK1

	**Overall survival**		
	**RR**	**95% CI**	***P*-value**
*Age at diagnosis*			
Per year	1.087	1.039–1.123	<0.001
			
*FIGO stage*			
I	1.000		
II	1.065	0.222–5.110	0.006
III	1.448	0.389–5.384	
IV	10.759	2.130–54.360	
			
*Grade*			
G1	1.000		0.051
G2	4.432	0.935–20.999	
G3	7.108	1.427–35.409	
			
*PLK1 expression*			
Negative	1.000		0.030
Positive	2.413	1.087–5.356	

RR=relative risk; CI=confidence interval; PLK=Polo-like kinase; IRS=immunoreactivity-scoring system.

) with a *P*-value of 0.03. Patients whose tumours showed high PLK1 expression had a 2.4-fold risk of dying (confidence interval: 1.1–5.4) in the given time interval compared to patients with tumours expressing low amounts of PLK1.

## DISCUSSION

Since the first description of *Drosophila* gene *Polo* 15 years ago (Sunkel and Glover, 1988), PLK isoenzymes have been the focus of intense investigative efforts. While early works in this field concentrated mainly on the identification of Polo homologues in different species and on the role of this enzyme family in normal tissue development and mitosis, the focus of interest has changed towards the functional and prognostic role of PLK isoenzymes in human malignancies.

The central role of PLK isoenzymes in tumorigenesis has been emphasised lately by studies showing that PLK1 inhibition, either by antisense or siRNA, leads to dramatic antiproliferative effects on tumour cells *in vitro* (Spänkuch-Schmitt *et al*, 2002a, 2002b; Elez *et al*, 2003; Liu and Erikson, 2003), pointing at a potential therapeutic use of inhibitory strategies targeting PLK isoenzymes. In-depth expression analysis of members of the PLK family in various cancers and correlation with other tumour characteristics may thus provide the translational basis for such approaches in clinical practice.

In earlier studies, we and others found PLK1 overexpressed in a variety of tumours, compared to the respective corresponding tissue of origin, suggesting an important role of high levels of PLK isoenzymes for tumour growth. For PLK isoenzymes other than PLK1 only sparse information on expression in various tumour entities is available, with only one study in mice reporting a relative loss of expression for PLK3 mRNA in colon carcinomas in comparison to adjacent normal colon mucosa (Dai *et al*, 2002).

In this study on epithelial ovarian tumours, we observed that normal ovarian surface epithelium as well as ovarian stroma does not express PLK1 or PLK3. The expression of PLK1 and PLK3 was elevated in a fraction of cystadenomas and was even higher in the group of primary ovarian carcinomas, whereas borderline tumours tended to express lower levels of PLK1 and PLK3 compared to other epithelial tumour entities of the ovary.

This finding might be in line with the concept that serous borderline tumours of the ovary should not be placed as an ‘intermediate lesion’ between benign cystadenomas and invasive carcinomas (Dietel and Hauptmann, 2000), but should be regarded as an independent tumour entity.

An earlier study carried out on 17 cases of ovarian carcinoma reported an overexpression of PLK1, which correlated positively with tumour stage and tumour grade (Takai *et al*, 2001b). In our expanded study population we were not able to reproduce these correlations.

However, we have shown that PLK1 overexpression has independent prognostic significance in ovarian carcinomas. This hypothesis matches well with observations that PLK1 expression is a survival parameter in oesophageal carcinoma (Tokumitsu *et al*, 1999), lung carcinoma (Wolf *et al*, 1997) and squamous cell carcinoma of head and neck (Knecht *et al*, 1999), and serves as a marker for metastatic disease in malignant melanoma (Kneisel *et al*, 2002).

With respect to its function, there is convincing evidence that PLK1 is essential in G2/M-phase transition of both normal and malignant cells. This isoenzyme is able to activate CDC25c which in turn activates the CDC2/Cyclin B1 complex leading to the import of Cyclin B1 into the nucleus (Toyoshima-Morimoto *et al*, 2002). Moreover, a recent study suggests that PLK1 is able to phosphorylate Cyclin B1 (Jackman *et al*, 2003) directly, and that specific phosphorylation cooperated with other signals in the nuclear import of Cyclin protein, a crucial checkpoint for the initiation of mitosis (Yuan *et al*, 2002). Later in mitosis, PLK1 has been implicated in the regulation of the anaphase-promoting complex (Kotani *et al*, 1998; Golan *et al*, 2002), centrosome maturation and destructions of cohesins (Sumara *et al*, 2002).

Data for PLK3 function is sparse and somewhat contradictory. Some authors suggested that PLK3 protein levels and kinase activity peak in late S and G2 phase (Ouyang *et al*, 1997; Chase *et al*, 1998), while other authors did not find any change of PLK3 expression throughout the cell cycle (Bahassi el *et al*, 2002).

The importance of PLK isoenzymes for mitosis in ovarian carcinoma is underlined by our finding that the expression of both PLK1 and PLK3 correlated with the mitotic activity. Nevertheless, it is surprising that only part of the variability of PLK isoform expression in our study population can be explained by variation in the number of mitotic figures, which might in part be due to technical difficulties in identifying mitotic cells in fixed tissue specimen.

On the other hand, one possible explanation might be that apart from the suggested role in mitosis, there might exist additional functions of PLK isoforms in intracellular signal transduction pathways. It has been reported that PLK3 is overexpressed in adherent *vs* nonadherent macrophages, and that it interacts with calcium- and integrin-binding protein (CIB), a small protein involved in integrin signalling pathways (Holtrich *et al*, 2000). As PLK3 is also expressed in irreversible postmitotic cells like neuronal ganglion cells (data not shown), it is intriguing to speculate on the basis of data obtained in neuronal cells (Kauselmann *et al*, 1999) and in macrophages (Holtrich *et al*, 2000) that the proposed interaction of PLK3 with CIB might play a role in determining the adhesiveness of tumour cells, albeit functional evidence for such a role of PLK3 in tumours is lacking.

Nevertheless, an additional functional role of PLK isoforms in determining adhesiveness and invasiveness of tumour cells might match with our observation that PLK isoform expression is pronounced at the leading edge of tumour invasion in ovarian carcinoma, which confirmed part of the observations made by Takai *et al* (2001b).

Taken together, we showed that PLK1 and PLK 3 are frequently overexpressed in highly proliferating malignant epithelial ovarian tumours and that overexpression is associated with mitosis and worse patients prognosis. We additionally hypothesised that PLK1 expression might serve as an independent prognostic factor in ovarian carcinomas. Further functional studies are needed to enlighten the possible role of PLK isoforms in the progression of these tumours and to answer the question as to whether the role of PLK isoforms in either mitotic machinery or regulation of other signal transduction pathways, determining malignant cell behaviour, might define them as attractive targets for novel forms of directed tumour therapy.
